# Effect of Combined Mental Task and Metaboreflex Activation on Hemodynamics and Cerebral Oxygenation in Patients With Metabolic Syndrome

**DOI:** 10.3389/fphys.2020.00397

**Published:** 2020-05-14

**Authors:** Azzurrra Doneddu, Silvana Roberto, Virginia Pinna, Sara Magnani, Giovanna Ghiani, Gianmarco Sainas, Gabriele Mulliri, Stefano Serra, Seyed Alireza Hosseini Kakhak, Raffaele Milia, Romina Lecis, Marco Guicciardi, Antonio Crisafulli

**Affiliations:** ^1^Sports Physiology Laboratory, University of Cagliari, Cagliari, Italy; ^2^International PhD in Innovation Sciences and Technologies, University of Cagliari, Cagliari, Italy; ^3^Faculty of Physical Education and Sport Sciences, Hakim Sabzevari University, Sabzevar, Iran; ^4^Department of Pedagogy, Psychology, and Philosophy, University of Cagliari, Cagliari, Italy

**Keywords:** blood pressure, cardiovascular regulation, myocardial contractility, stroke volume, systemic vascular resistance

## Abstract

**Objective:** The hemodynamic response to muscle metaboreflex has been reported to be significantly altered by metabolic syndrome (MS), with exaggerated systemic vascular resistance (SVR) increments and reduced cardiac output (CO) in comparison to healthy controls (CTLs). Moreover, patients with metabolic disorders, such as type 2 diabetes, have proven to have impaired cerebral blood flow in response to exercise. Thus, we hypothesized that contemporary mental task (MT) and metaboreflex would result in reduced cerebral oxygenation (COX) in these patients.

**Methods:** Thirteen MS patients (five women) and 14 normal age-matched CTLs (six women) were enrolled in this study. All the participants underwent five different tests, each lasting 12 min: post-exercise muscle ischemia (PEMI) to activate the metaboreflex, control exercise recovery (CER), PEMI + MT, CER + MT, and MT alone. Cerebral oxygenation was evaluated using near-infrared spectroscopy with sensors applied to the forehead. Hemodynamics were measured using impedance cardiography.

**Results:** The main results show that MS patients had higher SVR and lower CO levels compared to the CTL group during metaboreflex activation. Stroke volume and ventricular filling and emptying rates were also significantly reduced. Moreover, when MT was added to PEMI, COX was significantly increased in the CTL group with respect to the baseline (103.46 ± 3.14%), whereas this capacity was reduced in MS patients (102.37 ± 2.46%).

**Conclusion:** It was concluded that (1) patients with MS showed hemodynamic dysregulation during the metaboreflex, with exaggerated vasoconstriction and that (2) as compared to CTL, MS patients had reduced capacity to enhance COX when an MT superimposed the metaboreflex.

## Introduction

Metabolic syndrome (MS) is constantly growing in prevalence worldwide and has reached epidemic proportions over the last two decades. Metabolic syndrome is associated with elevated incidences of circulatory abnormalities and cardiovascular events and is often the harbinger of type 2 diabetes mellitus (DM2). The International Diabetes Federation (2019) estimated that 20–25% of the global adult population suffers from MS.

In people with MS, hemodynamic response to exercise is altered, with signs of impaired left ventricular systolic and diastolic function and reductions in maximal stroke volume (SV), cardiac output (CO), and heart rate (HR; Dipla et al., 2010; Roberto and Crisafulli, 2017; Ladeiras-Lopes et al., 2018; Roberto et al., 2019). Peripheral vascular responsiveness defects and exaggerated vasoconstriction due to sympathetic over-activation were observed in response to effort (Fournier et al., 2014; Milia et al., 2015b; Limberg et al., 2016). Metabolic syndrome has also been associated with cognitive dysfunction and poor performance in processing speed, visuospatial ability, semantic fluency, and executive function (Yaffe et al., 2004; Segura et al., 2009; van den Berg et al., 2009; Yates et al., 2012; Guicciardi et al., 2019).

During exercise, the activity of the sympathetic nervous system (SNS) increases, leading to the recruitment of all the main hemodynamic modulators (i.e., chronotropism, inotropism, cardiac preload, and cardiac afterload) and elevated blood pressure (Nóbrega et al., 2014; Crisafulli, 2017). Sympathetic nervous system regulation is achieved by integrating the activity of the three main nervous mechanisms: the baroreflex, the central command (a feed-forward mechanism involving parallel activation of motor and cardiovascular centers), and the exercise pressor reflex (Rowell and O’Leary, 1990; Fisher and White, 2004; Boushel, 2010; Nóbrega et al., 2014; Crisafulli et al., 2015). The metabolic part of the exercise pressor reflex, commonly known as the “metaboreflex,” appears to be essential for a normal cardiovascular response to exercise (Strange et al., 1993; Amann et al., 2011; Nóbrega et al., 2014). In humans, hemodynamic abnormalities during the metaboreflex have been demonstrated in several cardiovascular and metabolic diseases, including heart failure, coronary artery disease, hypertension, DM2, obesity, and MS (Negrao et al., 2001; Rondon et al., 2006; Delaney et al., 2010; Dipla et al., 2010; Choi et al., 2013; Crisafulli et al., 2013; Greaney et al., 2014; Milia et al., 2015a, b; Magnani et al., 2018; Roberto et al., 2019). In all these conditions, arterial vasoconstriction is exaggerated and prevails over CO as the main mechanisms whereby the blood pressure response is achieved during the metaboreflex.

During exercise, cerebral blood flow (CBF) increases due to brain activation (Secher et al., 2008). The increment in oxygen delivery appears to be important since brain function deteriorates when cerebral oxygenation (COX) is reduced. Moreover, decrements in COX have been related to the development of central fatigue (González-Alonso et al., 2004; Rasmussen et al., 2010). Another important phenomenon recently reported in patients with metabolic disorders, such as DM2, is that CBF regulation is impaired during exercise. This dysregulation has been related to the elevated SNS drive, with reductions in COX and an associated reduction in central motor drive (Kim et al., 2015; Vianna et al., 2015). Since the metaboreflex enhances SNS activity, it is possible to hypothesize that the stimulation of this reflex leads to COX impairment in patients with MS. Moreover, we wondered whether metaboreflex-induced increments in SNS drive would oppose the CBF increase due to brain activity. Specifically, we wondered whether contemporary mental task (MT) and metaboreflex would result in reduced COX in these patients. Insofar as during exercise metaboreflex and MT are contemporaneously present, this phenomenon could be helpful from a physiopathological point of view as it may provide the basis for the poor predisposition to exercise often reported in individuals suffering from MS.

The use of metaboreflex to study COX instead of real exercise allows us to overcome the potential confusing factor related to the augmented brain activity due to motor cortex activation, which could overlap with the brain activity due to MT, thereby masking its effect on COX. Actually, metaboreflex activated by post-exercise muscle ischemia (PEMI) stimulates sympathetic tone without any motor cortex activation. In short, during the metaboreflex, the subject may experience a sympathetic activation similar to that of exercise, ruling out any interference due to motor cortex activity on COX. It should be considered that the mechanoreflex-mediated increase in sympathetic activity is also excluded during PEMI, although some level of redundancy with neural occlusion has been demonstrated between metaboreflex and mechanoreflex. That is, their effects do not sum (Nóbrega et al., 2014). Post-exercise muscle ischemia avoids technical difficulties related to data collection during exercise. In particular, during PEMI, the subject is seated stably without any excessive movement of the thorax. This allows a good estimate of hemodynamic parameters. Moreover, during PEMI, an increase in parasympathetic tone takes place, and this reduces chronotropism, but it does not affect contractility and arteriolar and venous tone as the vagus nerve does not significantly innervate, neither do ventricles and vasculature. Hence, during PEMI, there is a response in inotropism, preload, and afterload but not in HR (Iellamo et al., 1999; Crisafulli, 2017). Thus, the PEMI method is particularly useful to investigate the reserve in inotropism, preload, and afterload, as the reserve in chronotropism is ruled out (Crisafulli, 2017).

Starting from the above considerations, the aim of this study is twofold: (1) the first is to characterize the hemodynamic response and COX in patients with MS during the metaboreflex and (2) second is to discover whether the association of metaboreflex with mental stress would further impair hemodynamics and COX in these patients.

## Materials and Methods

### Study Population

Two groups of subjects were studied:

• MS group: 13 patients [five women, mean ± standard deviation (SD) of the mean of age 52.9 ± 11.2 years], who received a diagnosis of MS from at least a year (range 1–6 years). Patients were enrolled considering the definition of MS as proposed by the International Diabetes Federation Task Force on Epidemiological and Prevention National Heart, Lung, and Blood Institute, American Heart Association, World Heart Federation, International Atherosclerosis Society, and the International Association for the Study of Obesity (Alberti et al., 2009). Specifically, subjects who met three or more of the following five criteria were considered as being affected by MS: (1) waist circumference >88 and >102 cm for women and men, respectively; (2) high blood pressure (≥130/85 mmHg); (3) high triglycerides (≥150 mg/dl); (4) low HDL cholesterol (≤40 and ≤50 mg/dl in men and women, respectively); (5) and high fasting glucose level (≥100 mg/dl).
• Control (CTL) group: 14 age-matched healthy subjects (six women, mean ± SD of age 50.8 ± 8.1 years), without any known metabolic disease resulting from anamnesis and physical examination.

For both groups, the exclusion criteria were as follows: age ≤18 and ≥65 years, presence of any associated medical conditions that could interfere with the autonomic function and/or chronic cardiopulmonary diseases. Smokers and patients taking β-blockers, sympathomimetics, and/or tricyclic antidepressants were also excluded. All women were tested during the follicular phase of their menstrual cycle (i.e., within 10 days from the start of menstruation) as self-reported.

None of the subjects of the CTL group were under medical treatment at the time of the study, while eight patients of the MS group were on medication for high blood pressure levels and two for high cholesterol.

Written informed consent was obtained from all participants. The study was approved by the local ethical committee and conforms to the Declaration of Helsinki.

A recent publication conducted in the same participants of the present study shows psychological results of our experiment (Guicciardi et al., 2019).

### Experimental Design

After enrollment, subjects attended the laboratory on two different days: one was the preliminary baseline visit and one was the experimental session.

Specifically, during the preliminary visit, the participants underwent a medical examination with anamnesis, anthropometric measurement (height, weight, body mass index, waist circumference, and body composition), ECG, and blood pressure. Body composition [fat mass (FM), fat-free mass (FFM), and total body water (TBW)] was measured using the bioimpedance method, according to standard international criteria (Ibáñez et al., 2015) and using an impedentiometer (BIA 101, Akern, Florence, Italy). Blood samples were drawn for triglycerides, HDL cholesterol, and plasma glucose. After this preliminary screening, all participants performed a cardiopulmonary exercise test (CPT) on an electromagnetically braked cycle ergometer (CUSTO Med, Ottobrunn, Germany) to measure their physical capacity. The test consisted in a linear increase in workload (10 W/min), starting at 10 W, at a pedaling frequency of 60 rpm, until exhaustion, which was considered as the workload at which the subject was unable to maintain a pedaling rate of at least 50 rpm. A gas analyzer (ULTIMA CPX, MedGraphics, St. Paul, MN, United States), calibrated immediately before each test, was utilized to assess gas exchanges during exercise. Specifically, maximal oxygen uptake (VO_2__max_), maximum workload (W_max_), and maximum HR (HR_max_) were gathered. Achievement of VO_2__max_ was considered as the attainment of at least two of the following criteria: (1) a plateau in VO_2_ despite increasing workload (<80 ml/min); (2) a respiratory exchange ratio above 1.10; and (3) HR ± 10 beats/min of predicted HR_max_ calculated as 220-age. Finally, after the CPT, the subjects familiarized themselves with the staff, equipment, and procedures of the experimental session described in the next paragraph. This allowed them to get used to the environment and instrumentation. In this occasion, participants’ maximum handgrip strength was assessed as the peak reached during five maximal compressions on a hydraulic dynamometer (MAP 1.1, Kern, Balingen, Germany) in the non-dominant arm.

For the experimental session (the interval from the preliminary visit was at least 3 days, range 3–7 days), each participant reported to the laboratory and underwent the following study protocol:

1. PEMI session: 3 min of rest, followed by 3 min of exercise, consisting of rhythmic (30 compressions/min) dynamic handgrip in the non-dominant arm at 30% of the maximum assessed during the preliminary visit and using the same dynamometer. Exercise was followed by 3 min of PEMI on the exercised arm induced by rapidly (in less than 3 s) inflating an upper arm biceps tourniquet to 50 mmHg above peak exercise systolic pressure. The cuff was kept inflated for 3 min. Three minutes of recovery was further allowed after the cuff was deflated, for a total of 6 min of recovery. This protocol has been demonstrated to be able to study the metaboreflex in normal subjects as well as in patients since it traps muscle metabolites in the exercising limb and maintains stimulation of the metaboreceptors (Roberto et al., 2012; Crisafulli et al., 2013; Marongiu et al., 2013; Milia et al., 2015a; Magnani et al., 2018).
2. Control exercise recovery (CER) session: the same rest–exercise protocol used for PEMI was performed, followed by 6 min of CER without tourniquet inflation. The CER session was employed in order to create a control recovery situation without metaboreflex activation.
3. MT session: the MT was a computerized attentional interference test, called the Bivalent Shape Task (Esposito et al., 2013), aimed at testing the ability to suppress interference, implemented using the Psychology Experiment Building Language (PEBL). Psychology Experiment Building Language is a cross-platform open-source programming language designed to implement both simple and complex psychological tests. The MT lasted 3 min and required the participant to determine whether a shape at the center of the screen was a circle or a square by using a mouse moved with the dominant non-exercising hand. Visual response cues were shaded in either red or blue. In all cases, the color was irrelevant and not used to make the decision. The stimulus shape was presented in red, blue, or an unfilled black outline. Thus, three basic trial types were performed: congruent trials, in which the irrelevant color of the stimulus matched the response cue; neutral, in which the stimulus was black and white; and incongruent, where there was a mismatch between the (irrelevant) color and the response cue. An additional mixed trial was performed where all targets (congruent, incongruent, and neutral) were presented in random order. The test consisted in four consecutive test block types, preceded by a short practice block with one example of each type of stimuli. Each block contained 30 trials by default. The MT started after a rest period of 6 min and lasted for 3 min. Three minutes of further recovery was allowed after the end of MT.
4. CER + MT session: the same rest–exercise protocol used for CER was performed. The exercise phase was followed by an MT session of 3 min, that is, the same duration of the MT session described at point (3). Three further minutes of recovery was allowed after the termination of MT.
5. PEMI + MT session: the same rest–exercise protocol used for PEMI was performed. The exercise phase was followed by 3 min of contemporary PEMI and MT. Three minutes of further recovery was allowed after the termination of the PEMI + MT sessions.

**FIGURE 1:**
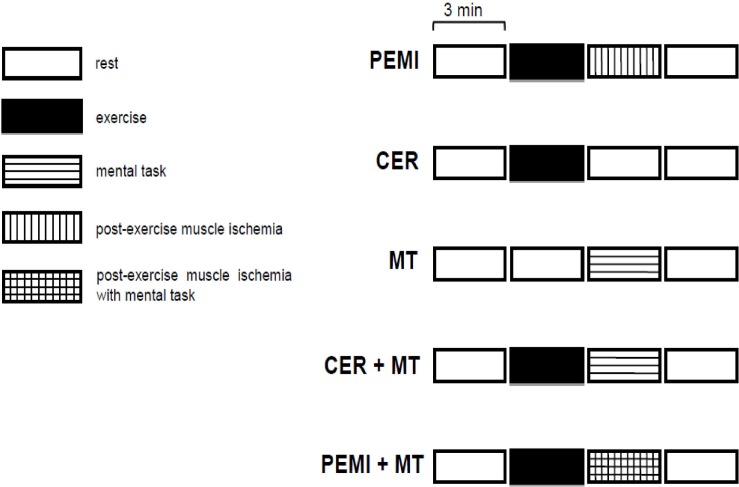
Schematic representation of the various sessions in the study protocol. PEMI, post-exercise muscle ischemia; CER, control exercise recovery; MT, mental task; CER + MT, control exercise recovery + mental task; PEMI + MT, post-exercise muscle ischemia + mental task.

The various sessions in the study protocol are schematized in Figure 1. All the sessions were composed of four blocks, each lasting 3 min, for a total of 12 min. Sessions were randomly assigned and were spaced by at least 15 min of recovery. Randomization was obtained using an online random sequence generator^1^. Recovery was considered complete when HR was not higher than 5 bpm compared to the pre-exercise level. Throughout sessions, subjects sat on a chair. All the experiments were carried out in a temperature-controlled, air-conditioned room (temperature set at 22°C and relative humidity 50%). Participants were requested to abstain from caffeinated beverages, alcohol, and heavy exercise for 12 h prior to reporting to the laboratory.

### Hemodynamic and COX Assessment

Subjects’ hemodynamic parameters were assessed throughout all the tests using the transthoracic impedance method, which assumes that changes in transthoracic impedance are representative of SV. By employing the Sramek–Bernstein equation, SV can be indirectly estimated (Bernstein, 1986). This kind of hemodynamic assessment has been previously used in several experiments dealing with the metaboreflex and has proven useful since it allows for a non-invasive hemodynamic estimate. The data acquisition and elaboration are described in detail in previous works from our laboratory (Crisafulli et al., 2008, 2009, 2013). Briefly, traces of electrocardiogram, thorax impedance (Z0), and Z0 first derivative derived from an impedance cardiograph (NCCOM 3, BoMed Inc., Irvine, CA, United States) were collected and stored using a digital chart recorder (ADInstruments, PowerLab 8SP, Castle Hill, Australia). Then, SV and HR were calculated beat to beat from stored traces. Cardiac output was obtained by multiplying SV by HR. The pre-ejection period (PEP) and the left ejection time (VET) were also measured using stored traces, as described in previous papers (Crisafulli et al., 2009; Sainas et al., 2016; Magnani et al., 2018; Roberto et al., 2019). Diastolic time (DT) was measured by subtracting the sum of PEP and VET from the total period of the cardiac cycle (Gledhill et al., 1994; Marongiu et al., 2013; Sainas et al., 2016), and the ventricular filling rate (VFR) was assessed by dividing SV by DT. Ventricular filling rate is a measure of the mean rate of diastolic blood flux (Gledhill et al., 1994; Crisafulli et al., 2009; Marongiu et al., 2013; Milia et al., 2015a). To obtain an index of myocardial performance, the mean systolic ejection rate (VER) was calculated as the SV/VET ratio (Gledhill et al., 1994; Sanna et al., 2017). Systolic blood pressure (SBP) and diastolic blood pressure (DBP) were assessed each minute by the same physician throughout all the protocol sessions using a standard manual sphygmomanometer placed in the non-exercising arm. Mean arterial blood pressure (MAP) was calculated by taking into consideration changes in the diastolic and systolic periods (Sainas et al., 2016). Systemic vascular resistance (SVR) was calculated as the MAP/CO ratio multiplied by 80, where 80 is a conversion factor to change units to standard resistance units.

Sympathetic activity toward the left ventricle was estimated by studying the PEP variations. Pre-ejection period has been demonstrated to be a useful inverse index of cardiac sympathetic activity with negligible influence of parasympathetic tone. Indeed, if there is more rapid development of intraventricular pressure, PEP shortens. This is normally the result of changes in sympathetic activity as ventricles are not innervated by para-SNS. Furthermore, PEP is not substantially altered by changes in HR (Michael et al., 2017).

Throughout the sessions, COX was assessed with near-infrared spectroscopy (NIRS; Nonin, SenSmart X-100, Plymouth, MN, United States), which provided a measure of oxygenated Hb in the brain tissue. The accuracy and reliability of the equipment were validated against a mix of 70% jugular bulb saturation and 30% arterial saturation (MacLeod et al., 2012). Cerebral oxygenation values are a representation of frontal cortical microcirculation in the gray matter, including arteries and veins. It is accepted that changes in COX are representative of cortical activation (Strangman et al., 2002a, b). This method has already been employed for COX assessment during MTs (Strangman et al., 2002a; Plichta et al., 2006; Verner et al., 2013). Two NIRS sensors were placed on the left and right sides of the subject’s forehead above the eyebrow, in the regions between Fp1 and F3 (international EEG 10–20 system) and adjusted according to the strength of the signal. The probes were covered and held in place using a headband and were taped to reduce the intrusion of extraneous light. Care was taken to ensure that the attached probes did not constrict the head and did not block circulation. Since the absolute concentration of oxygenated Hb could not be obtained because the path length of the NIRS light in the brain tissue was unknown, relative changes of NIRS signals against the baseline values were taken into consideration. Oxygenated Hb measured by NIRS may be considered an index of regional tissue blood flow as previously reported (Suzuki et al., 2004; Asahara et al., 2016; Ishii et al., 2018).

### Data Analysis and Calculation

Data are presented as means ± SD. The Kolmogorov–Smirnov test was used to assess distribution normality for each variable. Parametric tests were used for variables with normal distribution. To calculate the sample size required, we performed a power calculation using a power of 85%, an overall type 1 error of 0.05 (two-sided), and a 20% difference between groups in the studied variables. Twelve subjects were needed to obtain adequate statistical power. Differences between groups in anthropometric characteristics, levels of triglycerides, HDL cholesterol, plasma glucose, and results of the CPT test were found using the *t* test for unpaired data. The hemodynamic data collected during the five sessions of the protocol were averaged over 1 min. The differences between the groups in absolute values of hemodynamic data at rest and at the third minute of exercise (i.e., the sixth minute of the sessions) were tested using the two-way analysis of variance (ANOVA, factors: group and condition) followed by Bonferroni *post hoc* when appropriate. The same statistics were applied to the ninth minute of the sessions, which corresponded to the last minute of PEMI, MT, PEMI + MT, and CER + MT tests. In this way, the cardiovascular parameters were measured when a steady state was supposed to be reached. Two-way ANOVA (factors: group and condition) was also applied to changes in COX, which, as previously said, were reported as percentage changes from rest. Statistics were carried out using commercially available software (GraphPad Prism). Statistical significance was established as a *p* value of <0.05 in all cases.

## Results

The protocol was completed by all the subjects. The Kolmogorov–Smirnov test confirmed that distribution was normal for all the parameters examined; therefore, all the variables were analyzed using parametric tests. None of the participants reported unbearable pain or discomfort during the PEMI periods. The anthropometric characteristics of all the groups together with the results of the screening test and the CPT are shown in Table 1. The subjects in the MS group had higher weight, BMI, waist circumference, FM, SBP, DBP, blood triglycerides, and fasting glucose with respect to the CTL group. Table 1 also shows that FFM, TBW, HDL cholesterol, VO_2__max_, W_max_, and HR_max_ were lower in the MS group than in the CTL group.

**TABLE 1:** Anthropometric characteristics of groups together with results of the screening medical examination, cardiopulmonary test, and medication.

	CTL	MS	*p* value
Height (cm)	169.5 ± 10.29	165.92 ± 8.08	0.326
Body mass (kg)	69.45 ± 12.23	96.73 ± 14.13	<0.001
Body mass index (kg/m^2^)	24.03 ± 2.74	35.26 ± 5.65	<0.001
Waist circumference (cm)	80.75 ± 9.85	113.58 ± 7.24	<0.001
Fat mass (%)	21.8 ± 5.5	35.9 ± 7.3	<0.001
Fat-free mass (%)	78.2 ± 5.5	64.1 ± 7.3	<0.001
Total body water (%)	57.1 ± 4.5	47.3 ± 5.5	<0.001
Systolic blood pressure (mmHg)	111.07 ± 9.84	123.85 ± 8.69	0.001
Diastolic blood pressure (mmHg)	73.57 ± 6.91	81.15 ± 6.50	0.007
Triglycerides (mg/dl)	68.12 ± 19.94	139.85 ± 64.57	<0.001
HDL cholesterol (mg/dl)	63.28 ± 19.22	44.46 ± 14.74	0.008
Fasting glucose (mg/dl)	87.33 ± 9.92	103.92 ± 12.34	<0.001
Maximal O_2_ uptake (ml/kg/min)	31.70 ± 9.36	19.99 ± 3.46	<0.001
Maximum workload (W)	199.30 ± 84.39	140.76 ± 29.56	0.026
Maximum heart rate (bpm)	158.80 ± 12.45	147.84 ± 13.15	0.035
Sartans	0	4	
Calcium channel blockers	0	1	
ACE inhibitors	0	4	
Statins	0	2	

*CTL = controls (*n* = 14), MS = metabolic syndrome patients (*n* = 13). Values are mean ± SD.*

The data values recorded during the rest periods of the five protocol sessions are reported in Table 2. Statistics revealed that HR, SVR, and MAP were on average higher in the MS as compared to the CTL group, whereas SV, CO, and VER were lower. No group-related differences were found in VFR and COX. There was no condition effect for any of the studied variables, that is, the PEMI and CER tests started from similar conditions within the group. No significant interaction was found for any variable.

**TABLE 2:** Hemodynamic and cerebral oxygenation (COX) data values during rest periods preceding post-exercise control exercise recovery (CER), muscle ischemia (PEMI), mental task (MT), control exercise recovery + mental task (CER + MT), and post-exercise muscle ischemia + mental task (PEMI + MT) tests for control (CTL, *n* = 14), and metabolic syndrome (MS, *n* = 13) groups.

	CER	PEMI	MT	CER + MT	PEMI + MT	*p* value condition effect	*p* value group effect	*p* value interaction
HR (bpm)	CTL 65.88 ± 8.14	CTL 66.57 ± 8.43	CTL 67.36 ± 12.14	CTL 65.71 ± 9.49	CTL 66.99 ± 10.90	0.990	<0.001	0.989
	MS 75.02 ± 11.47	MS 75.02 ± 12.39	MS 73.55 ± 12.68	MS 72.95 ± 11.42	MS 74.16 ± 12.88			
SV (ml)	CTL 59.80 ± 21.88	CTL 58.03 ± 21.06	CTL 59.23 ± 21.48	CTL 58.90 ± 22.74	CTL 59.69 ± 21.23	0.974	<0.001	0.996
	MS 44.98 ± 11.01	MS 41.51 ± 7.88	MS 44.70 ± 15.50	MS 46.40 ± 13.99	MS 45.60 ± 15.71			
CO (L/min)	CTL 3.93 ± 1.48	CTL 3.82 ± 1.31	CTL 3.96 ± 1.50	CTL 3.78 ± 1.55	CTL 4.00 ± 1.55	0.985	0.002	0.997
	MS 3.33 ± 0.78	MS 3.11 ± 0.78	MS 3.20 ± 0.91	MS 3.33 ± 0.96	MS 3.28 ± 0.89			
VFR (ml/s)	CTL 117.79 ± 47.03	CTL 114.62 ± 41.11	CTL 121 ± 51.92	CTL 115.28 ± 49.14	CTL 122.04 ± 52.22	0.991	0.142	0.981
	MS 113.84 ± 32.43	MS 106.14 ± 40.20	MS 104.59 ± 38.22	MS 105.84 ± 41.19	MS 105.38 ± 34.25			
VER (ml/s)	CTL 230.64 ± 82.28	CTL 227.25 ± 85.74	CTL 233.78 ± 89.29	CTL 235.26 ± 81.34	CTL 233.17 ± 80.66	0.969	<0.001	0.998
	MS 181.88 ± 45.56	MS 170.43 ± 33.63	MS 181.18 ± 47.88	MS 189.04 ± 49.77	MS 181.16 ± 48.93			
SVR (dynes/s/cm^5^)	CTL 2,005.93 ± 862.87	CTL 1,991.08 ± 764.13	CTL 1,920.41 ± 702.57	CTL 1,985.61 ± 812.70	CTL 1,995.86 ± 863.13	0.997	<0.001	0.973
	MS 2,439.44 ± 753.67	MS 2,553.14 ± 736.80	MS 2,670.87 ± 1,010.41	MS 2,581.54 ± 904.53	MS 2,594.21 ± 832.17			
MAP (mmHg)	CTL 85.11 ± 8.77	CTL 84.19 ± 8.50	CTL 83.60 ± 8.05	CTL 83.34 ± 8.35	CTL 85.89 ± 8.06	0.662	<0.001	0.745
	MS 95.31 ± 9.85	MS 93.12 ± 8.30	MS 96.94 ± 9.93	MS 97.87 ± 6.88	MS 98.63 ± 9.05			
PEP (ms)	CTL 136.04 ± 16.02	CTL 137.52 ± 14.36	CTL 141.23 ± 19.50	CTL 139.52 ± 15.07	CTL 138.58 ± 25.11	0.834	0.744	0.791
	MS 145.27 ± 37.13	MS 142.02 ± 31.18	MS 144.51 ± 23.02	MS 134.37 ± 20.14	MS 133.66 ± 37.60			
COX	CTL 69.36 ± 5.81	CTL 68.15 ± 4.48	CTL 68.16 ± 4.91	CTL 68.86 ± 4.58	CTL 68.83 ± 4.56	0.995	0.206	0.985
	MS 69.71 ± 5.68	MS 69.92 ± 5.45	MS 69.90 ± 5.89	MS 69.66 ± 5.24	MS 69.92 ± 5.81			

*HR, heart rate, SV, stroke volume; CO, cardiac output; VFR, ventricular filling rate; VER, ventricular emptying rate; SVR, systemic vascular resistance; MAP, mean arterial pressure; PEP, pre-ejection period. Values are mean ± SD.*

Table 3 shows that the differences between groups in parameters found at rest were also present during the handgrip test, with the MS group showing higher HR, SVR, and MAP and lower SV, CO, and VER values than those of the CTL group. Moreover, MAP, PEP, and COX were significantly affected by the condition. In particular, in the CTL group, during the MT test (i.e., a condition when there was neither exercise nor MT at this time point in the protocol), MAP was lower than that during the CER + MT and the PEMI + MT tests, while in the MS group, MAP was lower during the MT test than during all the other protocol sessions. Moreover, in the CTL group, PEP was lower in the CER + MT than in the MT test. In both groups, COX increased during the CER, the PEMI, the CER + MT, and the PEMI + MT tests as compared to the MT test.

**TABLE 3:** Hemodynamic data values during the third minute of exercise (dynamic handgrip) of post-exercise control exercise recovery (CER), muscle ischemia (PEMI), mental task (MT), control exercise recovery + mental task (CER + MT), and post-exercise muscle ischemia + mental task (PEMI + MT) tests for control (CTL, *n* = 14), and metabolic syndrome (MS, *n* = 13) groups.

	CER	PEMI	MT	CER + MT	PEMI + MT	*p v*alue condition effect	*p* value group effect	*p v*alue interaction
HR (bpm)	CTL 70.75 ± 8.49	CTL 70.70 ± 7.55	CTL 67.19 ± 11.14	CTL 71.78 ± 10.15	CTL 72.12 ± 2.52	0.396	< 0.001	0.994
	MS 80.19 ± 12.04	MS 78.47 ± 9.35	MS 74.76 ± 12.13	MS 79.30 ± 12.94	MS 79.14 ± 11.64			
SV (ml)	CTL 62.56 ± 19.41	CTL 60.19 ± 18.78	CTL 58.24 ± 17.98	CTL 62.76 ± 25.10	CTL 60.26 ± 18.96	0.920	< 0.001	0.946
	MS 43.05 ± 9.42	MS 42.32 ± 11.03	MS 43.78 ± 14.04	MS 43.96 ± 12.89	MS 44.32 ± 8.78			
CO (L/min)	CTL 4.41 ± 1.26	CTL 4.25 ± 1.20	CTL 3.90 ± 1.33	CTL 4.50 ± 1.78	CTL 4.34 ± 1.43	0.879	0.003	0.888
	MS 3.42 ± 0.79	MS 3.29 ± 0.86	MS 3.20 ± 0.83	MS 3.40 ± 0.74	MS 3.48 ± 0.71			
VFR (ml/s)	CTL 113.42 ± 39.11	CTL 124.06 ± 39.55	CTL 119.29 ± 48.09	CTL 135.87 ± 63.30	CTL 126.46 ± 51.54	0.849	0.130	0.788
	MS 119.43 ± 41.53	MS 110.76 ± 37.86	MS 103.65 ± 33.42	MS 112.39 ± 31.77	MS 116.35 ± 32.33			
VER (ml/s)	CTL 208.98 ± 71.41	CTL 224.12 ± 68.69	CTL 224.86 ± 74.21	CTL 231.97 ± 81.87	CTL 214.63 ± 66.48	0.932	< 0.001	0.935
	MS 179.61 ± 36.90	MS 168.97 ± 35.91	MS 179.71 ± 39.84	MS 181.27 ± 42.46	MS 176.81 ± 38.07			
SVR (dynes/s/cm^5^)	CTL 2,128.70 ± 877.59	CTL 2,051.98 ± 741.54	CTL 1,962.31 ± 755.70	CTL 2,084.59 ± 893.77	CTL 2,186.34 ± 868.30	0.964	< 0.001	0.981
	MS 2,689.15 ± 724.85	MS 2,828.26 ± 899.34	MS 2,633.79 ± 945.56	MS 2,748.77 ± 691.66	MS 2,704.96 ± 737.14			
MAP (mmHg)	CTL 88.06 ± 8.78	CTL 92.99 ± 7.05	CTL 84.98 ± 6.65	CTL 94.26 ± 8.68^*^	CTL 95.48 ± 7.48^*^	< 0.001	< 0.001	0.507
	MS 109.24 ± 9.45^*^	MS 108.72 ± 10.83^*^	MS 97.28 ± 11.73	MS 111.42 ± 8.60^*^	MS 112.56 ± 10.70^*^			
PEP (ms)	CTL 125.20 ± 17.55	CTL 124.88 ± 18.91	CTL 141.20 ± 17.42	CTL 121.36 ± 19.29^*^	CTL 125.05 ± 16.17	0.008	0.818	0.989
	MS 128.31 ± 26.10	MS 126.71 ± 17.33	MS 140.20 ± 22.40	MS 123.44 ± 23.35^*^	MS 123.72 ± 25.30^*^			
COX (% of rest)	CTL 102.79 ± 1.52^*^	CTL 102.84 ± 1.98^*^	CTL 100.79 ± 1.41	CTL 102.61 ± 1.56^*^	CTL 103.3 ± 2.09^*^	< 0.001	0.969	0.524
	MS 102.6 ± 1.70^*^	MS 102.86 ± 2.02^*^	MS 100.36 ± 1.10	MS 103.10 ± 2.89^*^	MS 102.54 ± 1.27^*^			

HR, heart rate, SV, stroke volume; CO, cardiac output; VFR, ventricular filling rate; VER, ventricular emptying rate; SVR, systemic vascular resistance; MAP, mean arterial pressure; PEP, pre-ejection period; COX, cerebral oxygenation. Values are mean ± SD. * *p* < 0.05 vs. MT test of the same group.

**FIGURE 2:**
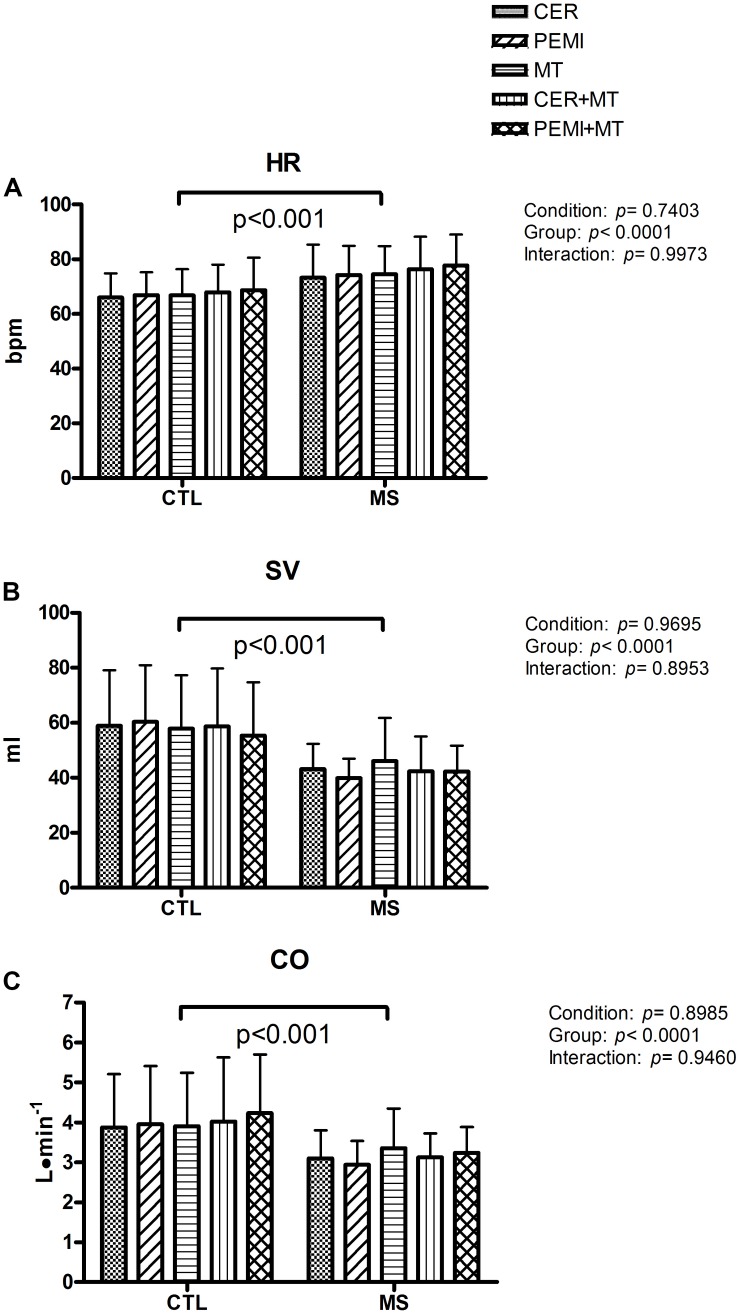
Hemodynamic data during control exercise recovery (CER), post-exercise muscle ischemia (PEMI), mental task (MT), control exercise recovery + mental task (CER + MT), and post-exercise muscle ischemia + mental task (PEMI + MT) tests in the metabolic syndrome (MS, *n* = 13) and the control (CTL, *n* = 14) groups. Data were gathered at the ninth minute of the sessions. HR, heart rate **(A)**; SV, stroke volume **(B)**; CO, cardiac output **(C)**. Values are mean ± SD. A horizontal bracket indicates the overall main effect of the groups.

**FIGURE 3:**
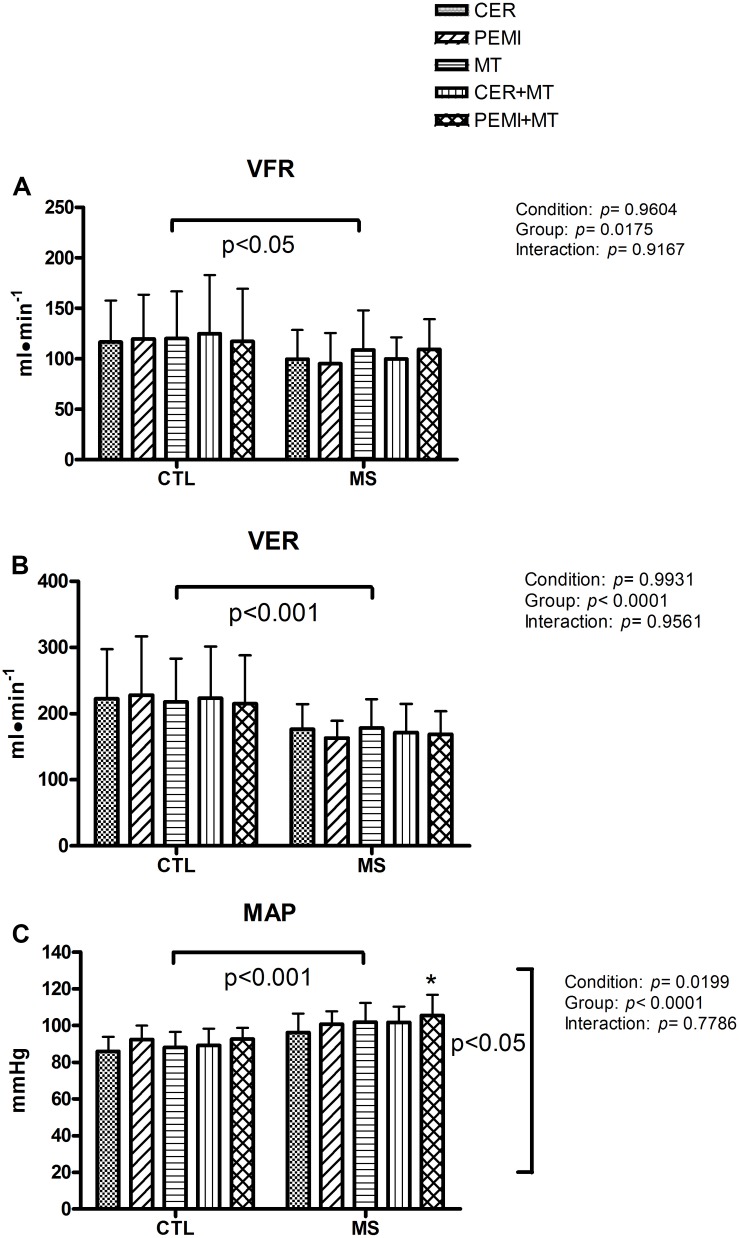
Hemodynamic data during control exercise recovery (CER), post-exercise muscle ischemia (PEMI), mental task (MT), control exercise recovery + mental task (CER + MT), and post-exercise muscle ischemia + mental task (PEMI + MT) tests in the metabolic syndrome (MS, *n* = 13) and the control (CTL, *n* = 14) groups. Data were gathered at the ninth minute of the sessions. VFR, ventricular filling rate **(A)**; VER, ventricular emptying rate **(B)**; MAP, mean arterial pressure **(C)**. Values are mean ± SD. A horizontal bracket indicates the overall main effect of the groups; a vertical bracket indicates the overall main effect of the condition. *p < 0.05 vs. CER test of the MS group.

**FIGURE 4:**
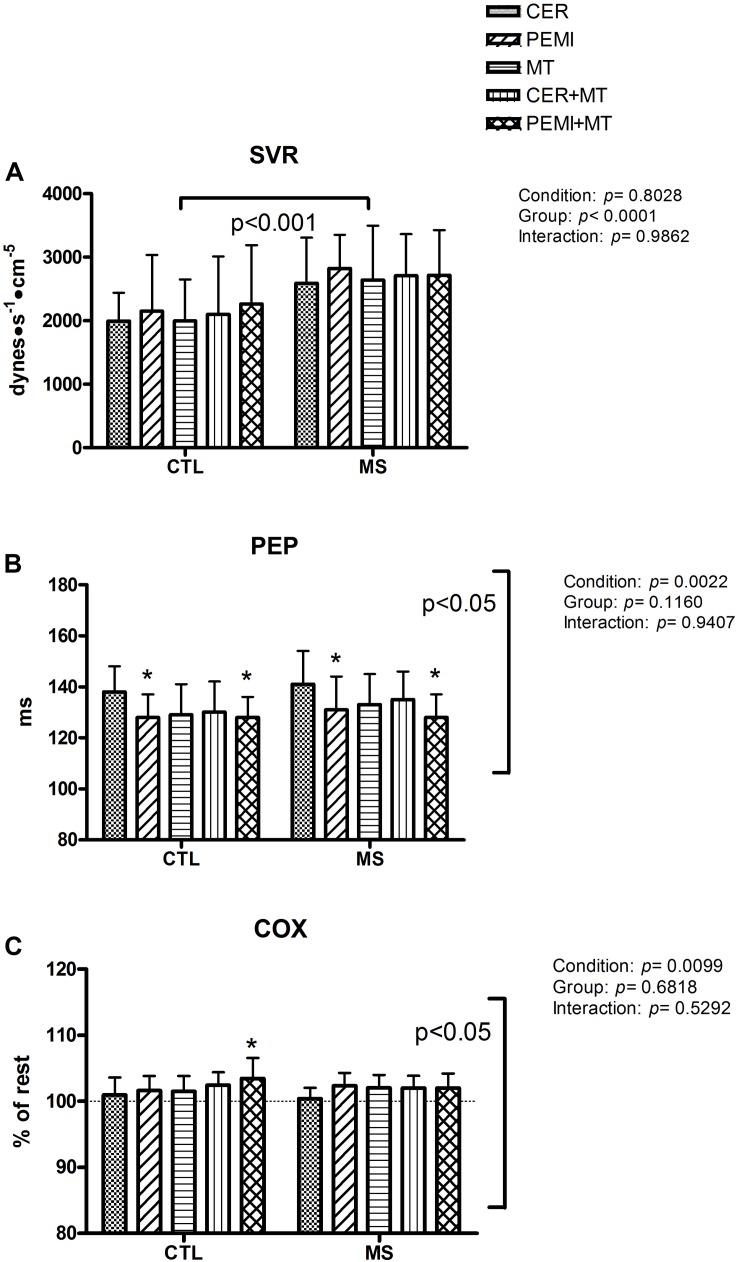
Hemodynamic data during control exercise recovery (CER), post-exercise muscle ischemia (PEMI), mental task (MT), control exercise recovery + mental task (CER + MT), and post-exercise muscle ischemia + mental task (PEMI + MT) tests in the metabolic syndrome (MS, *n* = 13) and the control (CTL, *n* = 14) groups. Data were gathered at the ninth minute of the sessions. SVR, systemic vascular resistance **(A)**; PEP, pre-ejection period **(B)**; COX, cerebral oxygenation **(C)**. In **(C)**, a dotted line indicates the rest level. Values are mean ± SD. A horizontal bracket indicates the overall main effect of the groups; a vertical bracket indicates the overall main effect of the condition. *p < 0.05 vs. CER test of the same group.

Figures 2–4 show the values of the cardiovascular parameters gathered at the ninth minute of recovery during the sessions, that is, when a steady state in variables was supposed to have been reached in response to the PEMI and the MT maneuvers. In detail, Figure 2A shows that HR was on average higher in the MS than in the CTL group. There was no difference arising from the condition, nor was there any HR difference among the various protocol sessions. Figure 2B demonstrates that SV was lower in the MS in comparison to the CTL group, without any detectable effect caused by the condition. As for HR and SV behavior, CO (Figure 2C) was lower in MS as compared to the CTL group. The condition did not lead to any statistical differences. No interaction effect was found for any of the variables.

Figure 3 illustrates that VFR (Figure 3A) and VER (Figure 3B) were on average higher in the CTL compared to the MS group. There was no condition or interaction effect. Figure 3C shows that MAP was higher in patients with MS compared to CTL subjects. Moreover, the statistics discovered a condition effect. *Post hoc* analysis revealed that in the MS group, MAP was higher in the PEMI + MT compared to the CER session (a condition where there was neither exercise nor MT at this time point in the protocol). There was no interaction effect for any of the variables.

Figure 4A exhibits that on average in the MS group, the SVR level was higher than that in the CTL group. There was no difference between the groups in terms of PEP (Figure 4B). However, the statistics turned up a significant condition effect. Through *post hoc* analysis, it was discovered that in both groups, PEP was lower in the PEMI than in the CER test. Furthermore, in both groups, PEP was lower in the PEMI + MT than in the CER test. Finally, Figure 4C shows that there was no difference in the COX (calculated as a percentage variation from rest level) between the groups. However, there was a significant condition effect. *Post hoc* analysis revealed that in the CTL this parameter was higher in the PEMI + MT than in the CER session, a situation where neither exercise nor MT was performed. No interaction effect was detectable for any of the parameters.

## Discussion

The first purpose of this study was to characterize the hemodynamic response and COX in patients suffering from MS during the metaboreflex activated using the PEMI method. We found that individuals with MS had altered hemodynamics during the PEMI-induced metaboreflex, as statistics found out a group effect for many of the hemodynamic parameters. In detail, on average, the subjects in the MS group showed higher MAP in comparison to the normal CTLs throughout experiments. Moreover, SV was lower, which was not compensated by elevated HR, resulting in CO that was lower than that in the CTL group. Thus, patients with MS reached the target MAP by means of an exaggerated SVR response. This hemodynamic scenario is not new and closely resembles the findings of other studies dealing with metaboreflex and MS (Milia et al., 2015b). It should be highlighted that in this study, hemodynamics was already altered at rest in patients with MS, thereby suggesting that the described cardiovascular alterations (i.e., reduced SV and CO and exaggerated SVR) were present even when metaboreflex was not stimulated. Thus, a group effect was present throughout experiments. It is also to be highlighted that in the CTL group, although on average the CO was higher than in the MS, we did not find a clear CO increment during the PEMI with respect to the CER session. This was different from what was reported in previous papers by our lab when the metaboreflex was investigated in younger individuals (Crisafulli, 2017). Rather, this response was closer to that described in older subjects, who reached the target MAP mainly by arteriolar vasoconstriction (Milia et al., 2015a).

As far as COX is concerned, for both groups in the study, COX increased to a similar extent when exercise was performed, that is, during the handgrip phases of the CER, the PEMI, the CER + MT, and the PEMI + MT test (about a 2–3% increase with respect to the baseline, as shown in Table 3). This increment, albeit small, was significantly higher as compared to the same time point in the MT session, when subjects were engaged neither in exercise nor in MT, as they were simply sitting in a chair (see the protocol details shown in Figure 1). Moreover, during the PEMI maneuver, there were undetectable differences between groups. Collectively, the results demonstrate that there was no difference between the groups in their ability to enhance COX either during the handgrip test performed at mild intensity or during the metaboreflex obtained using the PEMI maneuver. Therefore, the findings do not support the hypothesis that metaboreflex activation could lead to COX impairment in MS patients.

The second aim of the present investigation was to discover whether the association of metaboreflex activation with MT could impair COX in patients with MS. We found that, in comparison to the healthy CTLs, patients suffering from MS were unable to increase COX when MT was superimposed on the PEMI-induced metaboreflex activation. In particular, when we look at Figure 4C, it can be gleaned that in both groups, COX returned to the baseline during the recovery phases of the CER, the PEMI, the CER + MT, and the PEMI + MT tests, that is, after the handgrip strain ceased. At the same time point in the MT test, COX tended to slightly increase, although without statistical significance (101.48 ± 2.35% and 102.05 ± 1.95% with respect to the baseline in the CTL and MS groups, respectively). This mild MT-induced rise in COX was probably the consequence of the increased brain activity due to MT, which enhanced brain metabolism and led to CBF and COX elevation (Yaffe et al., 2004). Thus, it is possible to hypothesize that at this time point in the protocol, the COX level was similar across conditions due to the simultaneous COX increment in the MT test and the COX reduction in the CER, the PEMI, and the CER-MT tests.

However, there was one exception: during the PEMI + MT test of the CTL group, COX was significantly higher than at the same time point in the CER test of the same group (103.46 ± 3.14% and 100.98 ± 2.63% with respect to the baseline for the PEMI + MT and the CER tests, respectively). This shows that adding an MT to the PEMI maneuver increased COX in normal individuals. It should be noted that during this phase of the CER test, neither exercise nor MT was being performed (see Figure 1), as participants were sitting in a chair and recovering from the previous handgrip strain. Importantly, the PEMI + MT-induced COX increment described for CTL subjects was not present in the MS group (102.37 ± 2.46% and 100.40 ± 1.64% with respect to the baseline for the PEMI + MT and the CER tests, respectively). This occurrence suggests that patients suffering from MS were not able to increase COX to the same extent as CTL subjects when mental strain superimposed the PEMI-induced metaboreflex activation. Moreover, it would be expected that the MAP elevation occurring in the MS group during the PEMI + MT test would have caused an increase in COX. It is well known that blood pressure is one of the regulators of CBF and that raising MAP increases CBF and COX (Lucas et al., 2010; Willie et al., 2014). Thus, it may be expected that the MAP elevation which occurred in the MS group during the PEMI + MT test would have caused an increase in COX compared to the other protocol conditions. However, this was not the case. It should also be noted that on average the MAP level of the MS was above the level of the CTL group throughout the experiments. However, COX levels were almost always similar for both groups. Hence, it seems that some phenomenon prevented the enhancement of CBF and COX in MS patients, despite their high MAP level. Thus, collectively, our results suggest that the two groups behaved differently during the contemporary metaboreflex and MT.

It remains to be explained why COX increased in the CTL group during the PEMI + MT with respect to the CER test. In our opinion, this phenomenon was the consequence of the sum of the slight COX increments caused by both the PEMI maneuver and by MT engagement. The possibility of PEMI inducing an increase in CBF, and so in COX, has been already demonstrated and attributed to MAP elevation, although the exact physiological mechanisms remain unclear (Sander et al., 2010; Prodel Balanos et al., 2016; Ogoh et al., 2019). Actually, in the present study, there was a PEMI-induced COX increment in both groups (101.64 ± 2.17% and 102.36 ± 1.96% with respect to the baseline for the CTL and the MS groups, respectively). Similarly, during the same time point in the MT test, there was a slight increment in COX, and as previously pointed out, this was likely the consequence of an increase in brain metabolism due to brain activation. It appears as though in the CTL group, during the PEMI + MT test, the little COX increments due to both PEMI and MT summed, so that COX reached a level significantly higher than that in the CER test.

We cannot provide any conclusive explanation for why patients of the MS group appeared to have an impairment in COX during contemporary metaboreflex and MT. One possibility is that the individuals with MS had an elevated SNS drive which impaired their capacity to enhance CBF and COX. The cerebrovasculature is extensively innervated by adrenergic fibers which can potentially constrict brain circulation, even though the capacity of SNS to modulate CBF has not been precisely determined so far (Willie et al., 2014). Moreover, our data do not appear to support the presence of exaggerated sympathetic tone in MS patients, as testified by the PEP behavior. This parameter was similar in both groups and shortened to a similar extent in both groups in response to the PEMI and the PEMI + MT tests as compared to the CER session. PEP shortening has been demonstrated to be inversely related to sympathetic activity (Michael et al., 2017). Pre-ejection period is the time spent by the left ventricle to develop the amount of pressure necessary to overcome aortic pressure and allow the aortic valve to open. Since SNS activation enhances cardiac performance, during sympathetic stimulation, intraventricular pressure increases more rapidly (Michael et al., 2017). Moreover, PEP is not influenced by parasympathetic tone as ventricles are not innervated by the para-SNS. Thus, the similar PEP response for both groups seems to suggest that in MS patients, there was no exaggerated sympathetic activation. However, it should be underscored that, already at rest and throughout all the phases in the protocol, the MS patients’ HRs were higher than the CTL groups’ and that HR is directly related to SNS tone (Joyner, 2016). This suggests that SNS tone was always higher in the patients as compared to that in the CTLs, although it should be taken into account that a lower vagal tone could also be responsible for this phenomenon. Furthermore, the MS group had higher SVR levels in comparison to the CTL group. Since arteriolar tone is directly related to SNS activity, this occurrence further supports the possibility that SNS activity was more elevated in these patients than in normal individuals.

Hence, while the PEP analysis suggested a similar SNS tone for both groups, this result was contradicted by the HR and SVR behaviors. We cannot solve these contradictory findings as only direct measurement of SNS activity would be able to discern whether or not SNS was more elevated in the MS than in the CTL group. However, this kind of measurement is somewhat invasive and not applicable in experimental settings such as the present one, with ongoing psychological stress. A potential explanation for these conflicting results could be that in the MS group, SNS was already more activated at rest, and this would support a failing circulation. This possibility has already been demonstrated in patients suffering from overt heart failure (Piepoli and Crisafulli, 2014). It is then possible that the patients with MS had exaggerated SNS response during the metaboreflex, and, in the longer term, this increased vasoconstriction and impaired cardiac function, thereby initiating a vicious circle which may become harmful as it caused persistent vasoconstriction with vascular and endothelial damage. Actually, the occurrence of abnormal vascular responsiveness and exaggerated vasoconstriction has been observed in patients with metabolic disorders such as MS and DM2 (Milia et al., 2015b; Roberto and Crisafulli, 2017; Dal Canto et al., 2019). It should be noted however that research on this topic is very scarce. Thus, the actual mechanism implicated in the cardiovascular abnormalities observed in MS during the metaboreflex is an open area for future investigations. As a matter of fact, the MS group had a lower CO level, which seemed to be a consequence of reduced SV that was not compensated by their higher HR. This reduced SV resembled something usually found in heart failure (Piepoli and Crisafulli, 2014), and it could be explained by some phenomena. The first is that MS patients had impaired myocardial contractility, as testified by their lower VER. This is coherent with the previous findings which showed reduced systolic function without overt signs of heart failure in these patients (Fournier et al., 2014; Milia et al., 2015b). A second potential explanation for the low SV in the MS group could be the reduced venous return and the impaired cardiac preload, as suggested by their lower VFR in comparison to the CTL group. These results are in line with the notion that metabolic disorders can be associated with diastolic dysfunction and abnormalities in left ventricular relaxation (Powell et al., 2006; Mahmud et al., 2009), though the described abnormal diastolic function is not unanimously reported (Schillaci et al., 2006). Third, it is also possible that the reduced SV could be directly related to the exaggerated vasoconstriction exhibited by these patients, although whether SV reduction was caused by exaggerated vasoconstriction or the exaggerated vasoconstriction was caused by the reduction in SV appears to be a chicken-and-egg problem which deserves further investigation.

In summary, it is possible to hypothesize that the combination of reduced systolic and diastolic functions hampered SV and triggered compensatory mechanisms leading to SNS activation already at rest, thereby reducing the possibility of highlighting any increases in SNS due to metaboreflex and/or MT engagement. A similar vicious circle—where impaired heart function triggers compensatory mechanisms including SNS activation, exaggerated vasoconstriction, and elevated MAP—has already been proposed to explain the disturbed hemodynamics in heart failure (Piepoli and Crisafulli, 2014). In the longer term, this vicious circle may become harmful as it causes persistent vasoconstriction with vascular and endothelial damage. It should be noted however that research on this topic is very scarce. Thus, the actual mechanism implicated in the cardiovascular abnormalities observed in MS is an open area for future investigations.

Another potential phenomenon which could prevent the enhancement of CBF and COX in MS patients may be that cerebral vascular reactivity, that is, the capacity for cerebral vasodilation, was diminished in these patients. Several clues have suggested that the capacity to vasodilate the cerebral circulation in response to physiological stimuli, such as hypercapnia, is impaired in individuals suffering from metabolic disorders. What causes this impaired vascular function has yet to be completely elucidated. Low nitric oxide bioavailability and reduced endothelium-dependent vasodilation have all been proposed, but there is still no conclusive proof, and this phenomenon is probably multifactorial (Birdsill et al., 2013; Harrell et al., 2013; Kim et al., 2015; Mellendijk et al., 2015; Hurr et al., 2017). Further research is warranted in this area to better elucidate the mechanisms underlying the deficit in cerebral vasodilation in patients suffering from metabolic disorders.

A final potential mechanism underlying reduced COX in MS patients could be that they simply were not able to maintain CO at the proper level, thereby challenging cerebral circulation. Actually, as previously pointed out, CO was lower in the MS than in the CTL group throughout all the experimental sessions.

Whatever the cause (increased SNS activity, impaired capacity for cerebral vasodilation, and reduced capacity to increase CO), results suggest that patients suffering from MS were unable to properly increase COX when an MT activity superimposed the metaboreflex obtained by means of a PEMI maneuver. This phenomenon suggests that these patients may suffer from a sort of impairment in the cerebral flow. This could be intriguing as it may provide a potential physiopathological basis for the poor predisposition toward exercise often reported in patients with MS, since metaboreflex and MT are both operating during exercise (Wasmund et al., 2002). Actually, the ability to simultaneously perform physical tasks and MTs is something we experience every day.

### Limitations of the Study

One possible limitation of this study is that SNS activity was not directly assessed. Instead, an indirect measure was used. Direct SNS activity is somewhat problematic in human studies as it requires the use of microelectrodes to perform microneurography of peripheral nerves (Joyner, 2016). This technique introduces additional stress for the subjects engaged in MTs and thus is not advisable for experimental settings such this one. A non-invasive alternative method may be HR variability, which however has many limitations. For example, it is largely affected by the parasympathetic tone (Joyner, 2016).

A further potential limitation of this study is inherent to the use of NIRS. Compared with other brain imaging techniques, such as functional magnetic resonance imaging and transcranial Doppler, NIRS has a few limitations. One is the relatively low spatial resolution. Near-infrared spectroscopy devices, such as the one employed in this study, are usually designed to be placed on the forehead, thereby making it impossible to detect changes in areas that are far from the monitored site. Thus, the changes in COX described herein may provide a limited window of brain activity during both the PEMI and the MT maneuvers. Furthermore, NIRS is potentially influenced by changes in scalp perfusion (Ghosh et al., 2010; Verner et al., 2013). NIRS does not directly monitor CBF. Nevertheless, changes in Oxy-Hb may reflect changes in regional tissue blood flow, and validation studies suggest that NIRS meets the criteria for a relative (trend) monitor. These potential disadvantages however are outweighed by some practical features, such as low sensitivity to artifacts due to movements, low invasivity, and its low procedural and maintenance costs.

We must acknowledge that we did not assess the partial pressure of arterial CO_2_ (PaCO_2_) during the experiments. It is well known that reduced PaCO_2_ causes cerebral vasoconstriction. Thus, we cannot rule out that hyperventilation-related PaCO_2_ reduction occurred in MS patients during the PEMI + MT test, and this may have caused vasoconstriction in the patients’ cerebral circulation, thereby reducing COX.

In the interpretation of our results, it should also be considered that patients in the MS group were taking medication. Although individuals taking drugs known to affect autonomic nervous functions were excluded, several patients were taking sartans, calcium channel blockers, ACE inhibitors, and statins. We cannot rule out that these drugs may have influenced their cardiovascular response to the PEMI and the MT maneuvers. However, to the best of our knowledge, to date, no one has reported that any of these drugs have any impact on hemodynamics and/or cerebral circulation during metaboreflex and/or during MTs.

The fact that in the present investigation hemodynamics and COX were assessed in response to PEMI and not during real exercise is to be highlighted. Further study with different experimental settings is needed to find out whether similar impairments are present also during classical dynamic efforts (running, cycling, rowing, etc.).

## Conclusion

In conclusion, the results of the present investigation provide evidence that in subjects suffering from MS, hemodynamics were altered during the PEMI-induced metaboreflex. In these patients, target blood pressure was achieved by increasing SVR rather than by increasing CO. This is because they had reduced SV, which was not compensated by their elevated HR. Moreover, patients with MS were able to enhance COX during handgrip tests and during metaboreflex activation. However, it appears that they could not properly enhance COX when an MT superimposed the metaboreflex. It was concluded that the association of metaboreflex with mental strain may impair cerebral circulation in these patients. Further studies are warranted to confirm the phenomenon and to clarify whether or not MS impairs the cerebral circulation during the combination of MT and metaboreflex. Further research is also warranted to clarify whether the described hemodynamics and COX dysregulation observed during the metaboreflex are present during real exercise.

## Data Availability Statement

The datasets generated for this study are available on request to the corresponding author.

## Ethics Statement

The studies involving human participants were reviewed and approved by University Hospital of Cagliari Ethics Board. The patients/participants provided their written informed consent to participate in this study.

## Author Contributions

AD, SR, MG, and AC made the original study design and discussed with the other authors. VP, SM, GG, GS, GM, SS, SK, RM, and RL performed experiments and analyzed data. All authors contributed to data analysis and interpretation, as well as to the drafting of the manuscript and read and approved the manuscript.

## Conflict of Interest

The authors declare that the research was conducted in the absence of any commercial or financial relationships that could be construed as a potential conflict of interest.

## Footnotes

1 https://www.random.org/sequences/
